# Re-evaluation and re-analysis of 152 research exomes five years after the initial report reveals clinically relevant changes in 18%

**DOI:** 10.1038/s41431-023-01425-6

**Published:** 2023-07-18

**Authors:** Tobias Bartolomaeus, Julia Hentschel, Rami Abou Jamra, Bernt Popp

**Affiliations:** 1 Institute of Human Genetics, University of Leipzig Medical Center, Leipzig, 04103 Germany; 2 Berlin Institute of Health at Charité - Universitätsmedizin Berlin, Center of Functional Genomics, Hessische Straße 4A, 10115 Berlin, Germany

**Keywords:** Genetics research, Consanguinity, Genetic testing, Neurodevelopmental disorders, Next-generation sequencing

## Abstract

Iterative re-analysis of NGS results is not well investigated for published research cohorts of rare diseases. We revisited a cohort of 152 consanguineous families with developmental disorders (NDD) reported five years ago. We re-evaluated all reported variants according to diagnostic classification guidelines or our candidate gene scoring system (AutoCaSc) and systematically scored the validity of gene-disease associations (GDA). Sequencing data was re-processed using an up-to-date pipeline for case-level re-analysis. In 28/152 (18%) families, we identified a clinically relevant change. Ten previously reported (likely) pathogenic variants were re-classified as VUS/benign. In one case, the GDA (*TSEN15*) validity was judged as limited, and in five cases GDAs are meanwhile established. We identified 12 new disease causing variants. Two previously reported variants were missed by our updated pipeline due to alignment or reference issues. Our results support the need to re-evaluate screening studies, not only the negative cases but including supposedly solved ones. This also applies in a diagnostic setting. We highlight that the complexity of computational re-analysis for old data should be weighed against the decreasing re-testing costs. Since extensive re-analysis per case is beyond the resources of most institutions, we recommend a screening procedure that would quickly identify the majority (83%) of new variants.

## Introduction

Large scale exome sequencing (ES) studies (Deciphering Developmental Disorders Study, 2017 [1]) have revealed considerable heterogeneity for sporadic neurodevelopmental disorders (NDD) with hundreds of genes affected by de novo variants. The NDD term is used to summarize intellectual disability (ID), developmental delay, and autism spectrum disorders (ASD). Despite the fact that current NDD cohorts with presumed recessive inheritance due to parental consanguinity are much smaller, there are more NDD genes with recessive inheritance (982 definitively associated genes with recessive inheritance vs. 527 genes with dominant inheritance, SysNDD [2]: https://sysndd.dbmr.unibe.ch/, accessed 2022-08-25). The proportion of underlying recessive disorders in consanguineous NDD cohorts can be as high as 81% [3], validating the efficacy of the traditionally chosen technique of autozygosity mapping in disease gene identification. On the other hand, de novo, X-linked, and compound heterozygous variants should not be overlooked in consanguineous families [3–5].

In NDD cohorts examined using ES, the diagnostic yield is roughly 31–53% [6], leaving 47–69% unresolved. Among the explanations are limitations of the targeted regions in ES or tertiary data analysis [7]. The knowledge systematized in public databases is constantly increasing. Each year more than 300 new gene-disease associations (GDA) are identified in general [8] and about 160 for recessive NDD (https://sysndd.dbmr.unibe.ch/EntriesOverTime) [2]. This knowledge can be automatically queried. With the recent developments in the standardization of variant interpretation, data sharing, availability of large control databases, evolved computational tools, and filtering strategies, re-assessing published NDD cohorts is intriguing because: 1) improved variant calling algorithms can demask previously concealed genomic variation; 2) diagnostic variants can be evaluated according to current standards; 3) GDAs can be confirmed or removed; and 4) previously unknown gene associations can be uncovered.

Based on these considerations and following the terminology and recommendations of the ACMG [9] we decided to systematically re-assess a cohort of 152 families published by some of us in 2017 using analysis steps designed to cover the above points. Our aim was to quantify the possible gains of different re-assessment levels, contrast them with their complexity, and recommend an iterative re-evaluation, re-analysis, and data-sharing scheme for NDD research screening studies. We also identify possible problems in re-analysing outdated sequencing data.

## Materials and methods

### Cohort and ethics statement

The analyzed cohort was previously described by Reuter and colleagues in 2017 [10]. 152 core families with parental consanguinity and at least one child with NDD were studied. The cases were screened for genetic causes of NDD using autozygosity mapping and ES. The last data evaluation was between 2015-06-01 and 2016-08-31. Reuter et al. analyzed exome data sequentially by multiple analysts using an in-house pipeline. The Medical Faculties of Bonn (33/08) and Erlangen-Nürnberg (Re.-No. 4572) approved this cohort study. Some participants received compensation for travel costs.

### Re-evaluation of previously published diagnostic variants

**Fig. 1:**
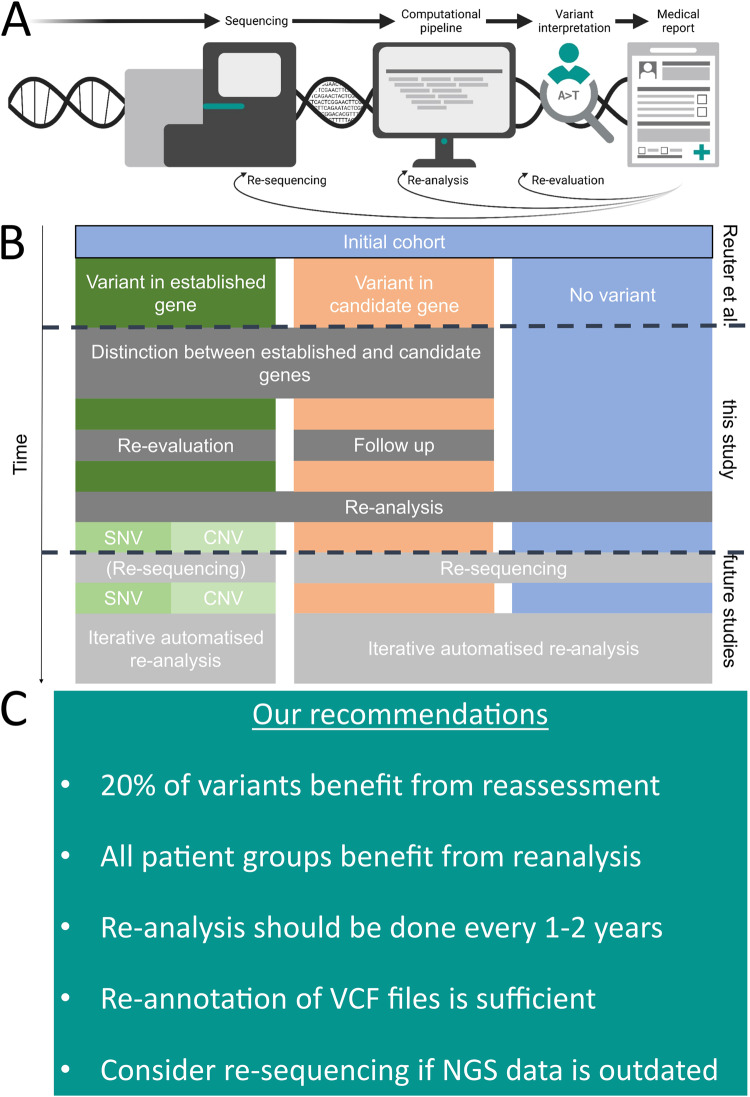
Infobox, re-evaluation and re-analysis flow diagram and recommendations. Panel **A** shows an infobox summarizing the basic terms in this study (panel created with BioRender.com). **B** A schematic flow diagram of the previous analyses performed by Reuter et al. on the same cohort, which we re-analysed with three of the main possible outcome columns from exome screening, performed the described analyses, and finally evaluated recommendations for future pipelines incorporating automated iterative re-analysis and re-sequencing. **C** Infobox with our recommendations for re-evaluating and re-analysing NDD screening studies.

Following the ACMG, we defined ‘re-evaluation’ as a new investigation and classification of previously reported variants [9]. For all variants previously reported by Reuter et al. in ‘known disease genes’, we evaluated the confidence in the GDAs based on the number of segregating variants, confirmatory publications, functional studies, and animal models. Next, all variants in genes with established phenotype associations were re-evaluated according to the ACMG classification [11] and the latest ACGS Best Practice Guidelines for Variant Classification (https://www.acgs.uk.com/media/11631/uk-practice-guidelines-for-variant-classification-v4-01-2020.pdf; Fig. 1; File S2 [12]). We submitted the results of our variant assessment to ClinVar [13] for all established GDAs.

### Follow-up of previously published variants in candidate genes

We then assessed the association and confirmation status of all variants in genes previously reported as NDD candidates by Reuter et al. (Fig. 1B; File S2 [12]). Despite ongoing attempts by the Clinical Genome Resource (ClinGen) [14] to standardize GDA strengths, there is presently no agreed definition or criteria for distinguishing between established and presumed GDAs. A GDA was considered established if it was reported in at least three families and by two research groups in separate publications. Alternatively, phenotype associations were recognized if only two families or one research group reported on this gene but functional data (defined as wet lab analyses interrogating the function of the affected gene product, excluding simple RT-PCR and western blot expression analyses, but including animal studies) was available. Genes associated with a phenotype in the literature but not reaching this level of confidence are called ‘published candidate genes’. These genes differ from ‘candidate genes’, which are only reported in large screening cohorts (see File S2 [12], column “Confidence_phenotype_association“ in variant tab). If a gene could not be reliably associated with an NDD phenotype, we used an in-house tool to score NDD candidate variants (AutoCaSc [15]; https://autocasc.uni-leipzig.de/; compare File S2 sheet “variants” for scoring results).

### Re-analysis of old sequencing data

In the context of our study, ‘re-analysis’ refers to the complete re-processing and evaluation of the sequencing data (Fig. 1A). We curated a list of all initial study sequenced individuals and families to collect and catalog alignment files (BAM) from archive harddrives. The BAM files were converted to unaligned FASTQ files, aligned to hg38, and called using a BWA/GATK pipeline to produce a multi-sample cohort VCF (variant call format) file. We annotated the file using up-to-date tools and databases and filtered it using custom scripts to produce variant lists for manual review. We also performed coverage-based copy number (CN) calling after clustering BAM files. The resulting variant lists were manually reviewed by an experienced geneticist, visualized using the IGV browser (https://software.broadinstitute.org/software/igv/) to assess variant quality, and evaluated for biological plausibility. Following that, the variants were evaluated in diagnostic and research settings. If needed, Sanger sequencing was used for segregation analysis. Method details are described in the Supplementary notes and Table S1.

## Results

### Cohort structure

**Fig. 2:**
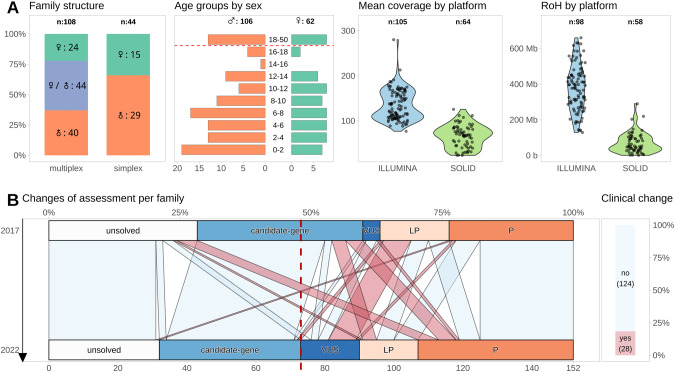
Cohort and data characteristics with results of re-evaluation. **A** Distribution of the 152 assessed families classified as simplex with one affected individual and multiplex with at least two affected individuals with sex distribution of the affected individuals in these groups (first panel). The age distribution by sex in the whole cohort (note, that not all individuals could be included in this plot since one index had no age and another had no sex data). The y-axis depicts age classes at 2-year intervals for pediatric cases, plus one class for adult index individuals. The x-axis shows the number of individuals, with females on the right (green) and males on the left (red) side (second panel). The mean coverage (third panel) and length of RoHs (fourth panel) were generally higher in samples analyzed by an Illumina platform than in samples sequenced with SOLiD technology, indicating quality differences. **B** The alluvial plot on the left side depicts the changes in clinical assessment conclusion of the families between 2017 and 2022 (Please note that this is distinct from variant classification changes. Reuter et al. identified, for instance, a variant in the candidate gene *CLMN* and a VUS in the *TRAPPC9* gene in the MR333 family. We reclassified the VUS as benign and identified a pathogenic variant in *ADNP* as the cause of the affected individual’s symptoms. The alluvial plot thus displays a line from VUS to P.). This results in clinically relevant changes in 28/152 families (18%, red; bar plot on right side colored according to the alluvial connections on the left side). The dashed line displays the shift in families where a VUS or (likely) pathogenic variant was identified. LP likely pathogenic, P pathogenic, RoH run of homozygosity, VUS variant of uncertain significance, ♀ female individual, ♂ male individual.

We collected sequencing data and information about age, sex, and phenotypes from 152 families (44 simplex with one, 79 multiplex with two, 24 with three, and five with four or more). The cohort characteristics are depicted in Fig. 2A (details in File S2 [12]). Most affected individuals were younger than 18 years (146/169 = 87%; one index without information). Generally, there were more affected males (male to female ratio = 107/62 ~ 1.7; one index without information). Males accounted for 29/44 (66%) simplex family cases. Most multiplex families (44/108, 41%) had affected individuals of both sexes, while 40/108 (37%) had only males and 24/108 (22%) had only females. The affected individuals originate from 15 countries. Previously, a trio-based ES method was used to discover putative de novo variations in 14/44 of families with only one affected individual (File S2 [12]; Reuter et al. stated 15/45, but we now regard MR309 as multiplex because of an affected paternal uncle).

### Availability and quality of sequencing

We collected at least one original ES file for 169/170 (99.5%) index cases and all parental trio samples. We couldn’t retrieve 14 BAM files sequenced on a Solexa platform in March/April 2010, but for 13 of these individuals, we had subsequent ES files, so only family MR061’s index lacked original files. We added 13 new BAM files for 11 affected individuals from 10 families where ES sequencing was performed post-publication for segregation analysis. In total, we collected 216 BAM files for re-analysis, 139 from Illumina, 76 from ABI SOLiD, and one from Ion Torrent (this file was excluded because we couldn’t devise a re-processing pipeline). For samples with multiple ES attempts, we chose the best BAM, resulting in 199 files (158 original index cases, 11 new affected siblings, and 30 parental).

Illumina files had significantly higher median mean read coverage (116; min: 77; max: 280) than SOLiD files (68; min: 24; max: 126) (Fig. 2A and File S2 [12]). The calculation from original hg19 to newly aligned hg38 BAMs took 17.1 CPU hours for SOLiD but only 2.9 hours for Illumina. Variant calling jobs defined by read count and covered regions took longer in Illumina BAMs (HaplotypeCaller 8.7 vs. 4.2 CPU hours). Lower coverage and quality of SOLiD files resulted in more variants remaining after filtering (median 28.5 vs. 10 homozygous calls), more CN calls (median 40.5 vs. 23.5), and more RoH calls (median 3.5 vs. 2.5); the summed RoH was shorter in the SOLiD samples (Fig. 2A), likely due to heterozygous artifact calls interrupting the regions.

Two previously reported *CBS* and *FAR1* variants were missed in the recalculated data, but more sensitive variant calling allowed the identification of a lowly covered homozygous *C12orf57* variant (see discussion and Figs. S3, S4 and S5).

### Novel diagnoses through re-analysis

Re-analysis revealed 12 (likely) pathogenic variants in 12 families (Table 1 and File S2 [12]). Seven (58%) of these novel 12 diagnoses were identified in previously undiagnosed 43 families (16% diagnostic rate). This includes heterozygous *ZEB2*, *HNRNPH2*, a hemizygous *TAF1*, and homozygous *DEGS1*, *YARS1*, *ESPN*, and *ADD3* variants.

**Table 1:** Previously undetected variants identified in re-analysis.

Family	Affected	Gene	Variant	Inheritance mode	Gene classification	Variant classification	Explains phenotype	Phenotype
MR152	2, m	*ADD3*	c.1100 G > A, p.(Gly367Asp)	AR	established	P (PS3,PM2,PM3,PP1_Moderate)	yes	ID
MR333	1, m	*ADNP*	c.2496_2499del, p.(Asn832Lysfs*81)	AD	established	P (PVS1,PS2_Strong,PM2)	yes	moderate ID, muscular hypotonia, gait disturbance, EEG abnormalities, cerebral atrophy
MR-SYR-14	2, fm	*ASXL3*	c.4462_4465del, p.(Thr1488Serfs*17)	AD	established	VUS (PVS1_Strong,PM2,BS3)	no	mild ID, microcephaly, aggressive behavior, self-mutilation
MR-DIV-01	2, m	*C12orf57*	c.1 A > G, p.0?	AR	established	P (PVS1_Moderate,PS4_Supporting,PM2,PM3,PP1_Strong)	yes	severe ID, seizures, muscular hypotonia, short stature
MR-SYR-49b	1, f	*DEGS1*	c.764 A > G, p.(Asn255Ser)	AR	established	P (PS3,PM2,PM3,PP1_Moderate)	yes	severe ID, cerebral atrophy
MR128^#^	1, m	*ESPN*	c.1916-1 G > C, p.0?	AR	established	P (PVS1,PM2,PM3_Supporting)	partially	severe ID, muscular hypotonia, deafness, strabismus, aplasia cutis congenita of scalp
MR-SYR-28	2, fm	*GCDH*	c.1204 C > T, p.(Arg402Trp)	AR	established	P (PS3,PS4_Supporting,PM2,PM3_Strong,PP4)	yes	very severe ID, seizures, muscular hypotonia, limb hypertonia, spasticity, short stature, microcephaly, leukodystrophy
MR136	1, f	*GRIN2A*	c.2077 A > G, p.(Asn693Asp)	AD	established	LP (PS2_Moderate,PM1,PM2,PP3)	yes	very severe, EEG abnormalities, muscular hypotonia
MR-TUR-05	1, f	*HNRNPH2*	c.616 C > T, p.(Arg206Trp)	AD	established	P (PS2_VeryStrong,PS4_Moderate,PM2)	yes	severe ID, myoclonus, microcephaly, muscular hypotonia, ataxia, EEG abnormalities
MR124	2, m	*SCN2A*	c.4606 A > G, p.(Ser1536Gly)	AD	established	VUS (PM2,PP3)	no	very severe ID, seizures, microcephaly, short stature, cataract, cryptorchidism, pyloric stenosis, cerebral atrophy, hypoplystic corpus callosum
MR-SYR-06	5, f	*SLC35A1*	c.508-6 T > C, p.?	AR	established	VUS (PM2)	no	profound ID, muscular hypotonia, cerebral atrophy, seizures, short stature
MR073	3, m	*TAF1*	c.2590 C > T, p.(Arg864Trp)	XL	established	LP (PM2,PP1_Moderate,PP2,PP3)	yes	moderate ID, mental deterioration, microcephaly, nystagmus
MR125	2, m	*YARS1*	c.1099 C > T, p.(Arg367Trp)	AR	established	P (PS4_supporting,PM2,PM3,PP1_Strong,PP3(accordingtounpublisheddata))	yes	ID
MR-SYR-49c	1, m	*ZEB2*	c.2177_2178del, p.(Ser726Phefs*29)	AD	established	P (PVS1,PM2,PP4_Moderate)	yes	severe ID, seizures, microcephaly, cerebral atrophy, hypoplastic corpus callosum, atrial septal defect, pulmonic stenosis
MR145	2, m	*ZMIZ1*	c.418 T > C, p.(Ser140Pro)	AD	established	VUS (PM2,PP3)	no	moderate ID, microcephaly, short stature
MR-DIV-02^§^	2, fm	*ZNF292*	c.3460_3463del, p.(Val1154Ilefs*7)	AD	established	LP (PVS1_Strong,PM2)	partially	mild ID, small for gestational age, short stature, microcephaly
MR-SYR-21	2, m	*ARHGEF6*	c.257 A > C, p.(Asp86Ala)	XL	weak candidate	not applicable	not applicable	severe ID, limb hypertonia, microcephaly, short stature
MR071a	1, f	*ZNF143*	c.44_45del, p.(Glu15Valfs*25)	AR	weak candidate	not applicable	not applicable	severe ID, muscular hypotonia, recurrent infections, microcephaly

*AD* autosomal dominant, *AR* autosomal recessive, *dn* de novo, *EEG* electroencephalogram, *f* female, *hemi* hemizygous, *het* heterozygous, *hom* homozygous, *ID* intellectual disability, *LP* likely pathogenic, *m* male, mat maternal, *n.a.* not available, *P* pathogenic, *pat* paternal, *VUS* variant of uncertain significance, *XL* X-linked; compare File S3 [12].

^#^: the pathogenic *ESPN* variant only explains part of the phenotype (deafness); ^§^: the *ZNF292* variant does not explain the microcephaly and short stature or the NDD phenotype in the other affected individuals from this family (see dual diagnosis results in Supplementary notes); *: both affected siblings inherited the *ASXL3* variant from the unaffected father.

Five of the 12 novel diagnoses (42%) were found in five of the 49 families with a candidate gene variant (Fig. 2B). Novel diagnoses include *ADNP*, *GRIN2A*, and *ZNF292* heterozygous variants and *GCDH* and *C12orf57* homozygous variants, reducing the plausibility of candidate genes (*BDH1*, *CLMN*, *FBXO11*, *FNDC3A*, *ENO2*). We likely clarified or added diagnoses in 10% of cases thought to be solved by a candidate gene.

In three families (MR124, MR-SYR-06, MR145), we found a VUS in *SCN2A*, *SLC35A1*, and *ZMIZ1*, respectively, but could not perform segregation analysis without parental samples. In a fourth family, MR-SYR-14, both affected siblings inherited a frameshifting variant in *ASXL3* from their unaffected father (Table 1, Fig. S6). This variant is located upstream of truncating variants in *ASXL3* reported to be pathogenic, and parental mosaicism is frequent [16]. Sanger sequencing on peripheral blood DNA indicated the variant as heterozygous, but cannot reliably discern higher-grade postzygotic events, and no tissue samples were available for the father. The healthy father’s segregation result wasn’t enough to exclude the variant, so it was classified as VUS.

### Plausibility of gene association

Reuter et al. reported 62 variants in 58 established genes in 61 families and 53 variants in 53 candidate genes in 49 families (in one family, they found a VUS and a variant in a candidate gene); 43 families had no variants. We re-evaluated all genes reported by Reuter et al. (Fig. 1A). Two genes were downgraded from established to candidate status (*TSEN15* and *KDM6B*, see Table 2). Four genes were previously referenced as candidate genes (*EZR*, *EDC3*, *EEF1D*, *NCAPD2;* see File S2 [12]) and a GDA has been published [17–20], including individuals from this cohort. However, evidence for GDA was insufficient when we re-evaluated, so we consider these six genes published candidate genes. Six former candidate genes (*TMTC3*, *GALNT2*, *SLC44A1*, *TMEM94, GRM7, PTRHD1*) now have enough evidence for GDA, so we labeled them as established genes (see File S2 [12] for all genes and changes in gene classifications).

**Table 2:** Clinical relevant changes in variant classification after re-evaluation.

Family	Affected	Gene	Variant	Inheritance mode	Gene classification	Variant classification	Explains phenotype	Phenotype
MR205	3, m	*DARS2*	c.228-12 C > G, p.?	AR	established (11 affected from 8 families, 2 publications)	B (BS1,BS2)	no	moderate ID, seizures, cerebral palsy, cerebral atrophy
MR154	2, m	*FOXRED1*	c.874 G > A, p.(Gly292Arg)	AR	established (8 affected from 7 families, >3 publications, functional analysis)	VUS (PM2,PM3,PP3)	no	moderate ID, seizures, muscular hypotonia
MR-SYR-04	2, f	*HACE1*	c.402+5 G > A, p.?	AR	established ( >14 affected from >6 families, >2 publications)	VUS (PM2,PM3_Supporting,PP3)	no	severe ID, ataxia, muscular hypotonia, recurrent infections
MR319	2, f	*MTHFR*	c.199 C > T, p.(Pro67Ser)	AR	established ( >20 affected from >20 families, >2 publications)	VUS (PM2,PM3_Supporting,PP3)	no	severe ID, microcephaly, abnormality of the optic nerve, EEG abnormalities, cerebral atrophy, leukodystrophy
MR081	2, m	*TRMT10A*	c.348 G > C, p.(Lys116Asn)	AR	established ( >6 affected from >3 families, >3 publications)	VUS (PM2,PM3_Supporting)	no	severe ID, microcephaly, short stature, behavioral abnormality, cerebral calcification
MR326	2, fm	*LINS1*	c.786_842del, p.(Arg263_Ser281del)	AR	established ( >7 affected from >3 families, >2 publications)	VUS (PM2,PM3_Supporting,PM4)	no	moderate ID, aggressive behavior, stereotypical motor behaviors, strabismus
MR-ER-31711	1, m	*UBE3B*	c.[1445 T > A;1616T > C], p.[(Leu539Pro;Leu482His)]	AR	established ( >9 affected from >7 families, >2 publications)	VUS (PM2,PM3,PP3)	no	severe ID, feeding problems in infancy, abnormalities of the face, submucous cleft palate, strabismus, deafness, hypoplastic corpus callosum, hydrocephalus
MR058	1, m	*SLC6A8*	c.644 A > G, p.(Glu215 Gly)	XL	established ( >10 affected from >9 families, >3 publications)	VUS (PM2,PM3_Supporting,PP3)	no	moderate ID, feeding problems in infancy, congenital megacolon
MR-SYR-34	3, fm	*ADGRG1*	c.64+5G > A, p.?	AR	established ( >12 affected from >12 families, >3 publications)	VUS (PM2,PM3_Supporting)	no	very severe ID, seizures, limb hypertonia, mental deterioration, deafness, cerebral atrophy
MR305	1, m	*PIGA*	c.1261 G > C, p.(Gly421Arg)	XL	established ( >14 affected from >7 families, >3 publications)	VUS (PM2,PM3_Supporting,PP3)	no	very severe ID, seizures, microcephaly, spasticity, abnormalities of the face, gingival hypertrophy, nystagmus, scaphocephaly, schizencephaly, leukodystrophy, basal ganglia calcification
MR092	2, m	*TSEN15*	c.346 C > T, p.(His116Tyr)	AR	published candidate (5 affected from 3 families, no functional analysis)	not applicable	not applicable	moderate ID, microcephaly

*AR* autosomal recessive, *EEG* electroencephalogram, *f* female, *hemi* hemizygous, *hom* homozygous, *ID* intellectual disability, *m* male, *mat* maternal, *VUS* variant of uncertain significance, *XL* X-linked; compare File S3 [12].

### Changes in variant classification

We re-evaluated the 62 variants previously classified by Reuter et al. as (likely) pathogenic or VUS. Of the 37 pathogenic variants identified in 36 families, 35 (94%) are still considered pathogenic (28/37; 76%) or likely pathogenic (7/37; 19%), whereas one variant was changed to benign (*DARS2*), one to VUS (*LINS*; Table 2; Fig. 2B). Of the 20 previously likely pathogenic variants, six were classified as pathogenic, five remained as likely pathogenic, eight were downgraded to VUS, and one was not classified because of an uncertain GDA (*TSEN15*). Regarding the five VUS reported by Reuter et al., no further evidence was found to upgrade any of these variants to (likely) pathogenic. One was reclassified as benign (*TRAPPC9*), and one was not classified because a recessive inheritance mode was not established (*KDM6B*; Fig. 2B and File S2 [12]). Of the previously reported variants, 29/62 (47%) were not in ClinVar.

### Candidate gene confirmation through matchmaking

The candidate genes identified by Reuter et al. were followed up. In 10 (19%) of the formerly 53 candidate genes an association to a NDD was meanwhile published (*GRM7*, *EZR*, *EDC3*, *EEF1D*, *TMTC3*, *GALNT2*, *SLC44A1*, *PTRHD1*, *TMEM94*, *NCAPD2*; File S2). For another 10 (19%) candidate genes, our upload in GeneMatcher [21] resulted in the first positive contact with other research groups. In five families with previous candidate genes (*BDH1*, *CLMN*, *FNDC3A*, *FBXO11, ENO*), a likely pathogenic variant was found, contradicting their causality for the individuals’ symptoms. For the remaining 29 (54.7%), GeneMatcher hasn’t led to contact with other researchers.

### Examples of lessons learnt

During our re-analysis, we found several potential pitfalls that we and other genetics labs can learn from. The following are three exemplary case studies.

Multiple diagnoses in one family: Family MR-SYR-49 is subdivided into three branches named 'a', 'b' and 'c' (Fig. S1A). One variant in the candidate gene *CEP76* was reported in the 'a' branch. Follow up of this gene led to collaboration with other groups (ongoing research). During re-analysis, we identified a homozygous pathogenic variant in *DEGS1* (Fig. S1B) in the affected girl from the 'b' branch. The GDA was recently published [22]. The affected boy from the 'c' branch has the autosomal dominant Mowat-Wilson syndrome due to a heterozygous pathogenic variant in *ZEB2* (OMIM #235730). To summarize, this is a large family in which three members have three unique diseases with varied inheritance patterns. The initial strategy focused on one common cause, which didn't work.

Missing the X-chromosomal inheritance pattern: A hemizygous, likely pathogenic *TAF1* variant segregated with ID and craniofacial dysmorphism in family MR073 (Fig. S1C, Table 1). We expand the mutational spectrum of this disorder by adding a *TAF1* variant that disrupts DNA interaction (Fig. S1D). Due to insufficient information in population data banks and challenges in classifying inherited X-linked variants, most *TAF1* variants have unknown significance [23]. This family shows how consanguinity can obscure alternative inheritance patterns. We couldn't classify this variant as pathogenic without segregation analysis, which emphasizes the need to store index, parent, and affected family DNA samples.

Identifying new variants and excluding old ones: The homozygous *TRAPPC9* variant in MR333, described previously as VUS, was reclassified as benign. A *CLMN* candidate variant has also been reported. Re-analysis revealed a heterozygous (de novo) pathogenic *ADNP* variant that causes Helsmoortel-van der Aa syndrome (OMIM #615873). This shows that re-analysis helps more than just families with negative results. It also highlights that de novo variants are a possible cause, especially in singleton cases despite parental consanguinity.

## Discussion

The objectives of this study were to maximize the output of our cohort of 152 consanguineous families with children who have NDD and to estimate the effort-benefit ratio so that we can make informed decisions regarding re-evaluation and re-analysis. Thus, we first re-evaluated all previously reported variants and revisited previously reported candidate genes. The raw ES data was then re-calculated, which produced a uniform callset, and then re-analysed to identify variants in known genes or novel candidate genes. For each assessment level, the added value in diagnosing new families and insights gained for variant curators can be contrasted with effort and pitfalls.

Re-evaluating 152 families resulted in clinically significant changes in 28 families (18%) due to reclassification of previously reported variants, published candidate genes, or previously undetected disease-causing variants (Fig. 2B, right panel; Tables 1 and 2). While we conclude that it is generally prudent to re-evaluate all data older than five years, additional questions remain: Should this be done for all cases, or should certain levels of analysis be prioritized? Is it worthwhile to completely re-calculate old data, or should new data be generated? What timeframes are adequate for the different levels?

Eleven (18%) of Reuter et al.’s 62 variants were reclassified clinically. The previously reported diagnostic yield for confirmed NDD genes must be retroactively adjusted from 37 to 30% (−7%). This is consistent with other groups' findings that 40% of older variants need reinterpretation [24, 25]. With the standardization and wider application of classification criteria, it’s less likely that this number applies to current variants. Still, it shows how important and necessary it is to re-evaluate. When variants should be re-interpreted depends on the classification used (e.g., original ACMG recommendations for Reuter et al.), but as a rule of thumb, one could recommend reinterpreting variants classified before the recent ACMG/ACGS updates in 2019/2020.

After re-analysing exome data, 12 missed (likely) pathogenic variants were found, raising the corrected diagnostic yield again from 30 to 41% (+11%). From 2010 to 2016, when the original datasets were annotated, public databases had less genetic information, such as variant frequency, reported pathogenicity, or GDA. To evaluate the many variants practically, the analysis was limited to multiple affected family members' linkage regions. Several of the missed variants are outside of these linkage regions or are within them but could not be prioritized due to the large number of rare homozygous variants in the respective family. In addition, after Reuter et al., more GDAs were published. When the majority of the data had been analyzed previously, the ’de novo paradigm’ in NDD [26] had only recently been proposed and was not as widely accepted as it is today. The five novel de novo and X-linked variants found in this study went beyond the first study of this cohort and were made possible by better variant and disease databases.

Eight of 12 novel-positive families' original annotated VCF files were available. Seven of them had the correct variant in the old data. In the eighth family (MR-DIV-01), the *C12orf57* variant was missing, likely due to poor variant quality not passing a software threshold. Considering the computational effort for the full re-analysis, a re-annotation of the VCF files with updates from gnomAD, HGMD, and ClinVar would have been sufficient to find pathogenic variants for 7/8 (88%) of these newly diagnosed families.

Although extensive re-analysis of NGS data beyond individual cases may seem impractical, we want diagnostic institutions and other research groups with access to larger screening cohorts to regularly re-analyse their published data. In contrast to our extensive re-analysis of all individual variants, a systematic re-analysis could be limited to the following steps: a) rare likely loss-of-function (LoF) variants, b) pathogenic variants in ClinVar/HGMD, and c) rare missense variants with a known pathogenic variant at the same amino acid position. This could allow computational filtering to replace time-consuming manual re-analysis as recently shown [27–29]. In our study, 10/12 (83%) novel pathogenic variants would have been found using this 3-step filtering method.

Compared to other recent studies of NDD in consanguineous cohorts, our 41% new diagnostic yield seems low (43.3%–85.0%) [24, 25, 30–35]. However, the wide range of diagnostic rates reflects the diversity of the cohorts studied in terms of included phenotypes and other inclusion characteristics like family structure, minimal number of affected individuals, and degree of consanguinity. Sequencing technologies and processing pipelines may also affect yield. Other groups described a share of causal CNVs between 1–8% [30, 33, 35], which could represent one factor of additional diagnoses. Although we called CNVs in our re-analysis, we found no new disease-causing CNVs in our cohort. This diagnostic gap is likely because the cohort was pre-analysed for CNVs before the Reuter study. Besides, the old data is probably too inhomogeneous (multiple sequencing techniques, machines, and runs throughout the project; compare Fig. S2 [12]) to have allowed detection of very small CNVs (e.g. deletion of only one exon) by coverage based methods. However, when comparing overall diagnostic yield with our results, inconsistent reporting of candidate genes is a much more important factor. In most publications, the boundary between established GDA and 'novel genes' is not clearly defined. When strictly excluding variants in candidate genes as per our definition, the diagnostic yield in other cohorts is between 19–45% [31, 35], leaving our study in the upper middle range despite its considerable heterogeneity in inclusion criteria and sequencing data quality.

In the overall view of the results of various groups [36–41], the re-analysis of exome data contributes to the clarification of 10–36% of cases. In the re-analysis of 33 consanguine families with more than two affected individuals, disease-causing variants were added in 48% (*n* = 16) [7]. The described biases in the comparison with other studies of NDD in consanguineous families are further exacerbated in the comparison of the additional yield in the re-analysis of exome data. The comparison with other reports is even more confounded by our approach of re-analysing not only unsolved cases from a defined cohort, but also putatively solved cases from which we removed over-classified variants and associations, which, to our knowledge, have not been published in this way before.

Re-sequencing for this cohort was beyond the scope of our work, but the problems we encountered with re-calculation suggest possible benefits. Aligning old sequencing data to hg38 can result in ambiguous read alignment and missing variants, as with the *CBS* missense variant c.341 C > T, p.(Ala114Val) (Fig. S3). Some of the previous sequencing was done using SOLiD platforms. This outdated data cannot always be mapped correctly with the current mapping tools (e.g. *FAR1* in-frame indel c.495_507delinsT, compare Fig. S4), leading to time consuming manual adaptation of pipelines, high computational requirements, and missed variants as well. Older research screening data is often inadequate compared to current diagnostic standards, which led to a costly validation analysis. Thus, when weighing re-analysis over re-sequencing, effort and potential problems with liftover of old target files or alignment of older read data, compatibility of new pipelines, and quality of old data should be considered. In retrospect re-sequencing, either with diagnostic grade ES or ideally using genome sequencing, would certainly have been useful in our cohort and would have provided the additional benefit of high quality CNV analysis and detection of non-coding variants.

Our study shows the value of revisiting NDD screening data. Other groups have confirmed the need for re-analysis of sequencing data for families with no diagnostic findings and re-evaluation of reported variants. This is usually done by separating positive families from those with reported candidate genes and negative families. This leads to path dependency, where presumed positive families are considered to be conclusive. We demonstrate here that all groups benefit from re-analysis and that considerations of diagnostic yield should not be separated from re-evaluation of the entire cohort.

Based on our experience, the most promising and practical step is re-analysing existing variant calls by annotating and filtering them with the latest information and re-evaluating such identified variants together with previously reported ones according to current standards and new literature. A sensible timeframe to perform this iterative step in research cohorts could be about one to two years. Stringent submission and updates of all reported variants in known genes into public databases like ClinVar can’t be stressed enough in this regard. In contrast, computationally expensive re-analysis steps, like new alignments and variant calling, seem to provide no justifiable gain and should be reserved for re-calculations to normalize and integrate a sequenced cohort into large public datasets. While re-sequencing was not directly investigated in our study, several markers suggest a technological 'tipping-point' (e.g. current exome enrichment kits sequenced with 150 bp paired-end reads) has been surpassed for some of the sequencing data in this NDD cohort. The next tipping points for genetics screening could be genome sequencing, long-read sequencing, and functional sequencing like RNA-seq and Methyl-seq.

Our results support the need to regularly re-evaluate all NDD screening cases using re-annotation and efficient filtering. Rare disease studies should also incorporate biobanking protocols to enable novel re-sequencing steps.

## Web resources

AutoCaSc: https://autocasc.uni-leipzig.de/

ClinVar: https://www.ncbi.nlm.nih.gov/clinvar/

gnomAD browser: http://gnomad.broadinstitute.org/

SysNDD database: https://sysndd.dbmr.unibe.ch/

GeneMatcher: https://genematcher.org/

### Supplementary information

File S1

## Data Availability

All data generated or analyzed can be found either at the publisher’s website or has been uploaded to Zenodo (Files S2, S3: 10.5281/zenodo.8046795).

## Code availability

All code is publicly available on GitHub (https://github.com/berntpopp/conNDD).
